# Combination-Based Biofunctional Coatings for Veterinary Biofilm-Associated Infections

**DOI:** 10.3390/antibiotics15070703

**Published:** 2026-07-19

**Authors:** Muhammad Hassan Khalid, Bilal Aslam, Sulaiman F. Aljasir

**Affiliations:** 1 Institute of Microbiology, Government College University Faisalabad, Faisalabad 38800, Punjab, Pakistan; thehassanmughal@gmail.com; 2 Department of Veterinary Preventive Medicine, College of Veterinary Medicine, Qassim University, Buraydah P.O. Box 52571, Saudi Arabia

**Keywords:** veterinary, coatings, nanoparticles, deposition techniques, implants, wounds, polymers

## Abstract

Biofilm-associated infections present a significant but insufficiently acknowledged problem in veterinary medicine because they result in persistent infections that lead to unsuccessful treatments and drive the growth of antimicrobial resistance throughout animal healthcare systems. The extracellular matrix of biofilms together with their resistance mechanisms make traditional antimicrobial methods ineffective against these structures. The development of combination-based biomaterial coatings represents an effective solution for biofilm control since these coatings combine different antimicrobial capabilities into one surface treatment. This review offers an in-depth evaluation of veterinary-focused combination-based coating systems which scientists developed to create solutions for catheterization, orthopedic, dental implant procedures, wound treatment and aquaculture infrastructure. This review also examines various coating methods to determine their effectiveness in creating surfaces that optimize antimicrobial delivery. However, the development of veterinary medical solutions faces major obstacles because the technology needs to overcome some key issues, which include maintaining stability through time, protecting animal health, preventing environmental harm and meeting regulatory standards. Overall, the use of multifunctional biomaterial functional coatings provides veterinary medicine with a revolutionary method to handle biofilm-related infections in animals, which decreases the need for antibiotics while improving infection control according to One Health principles.

## 1. Introduction

**Figure 1:**
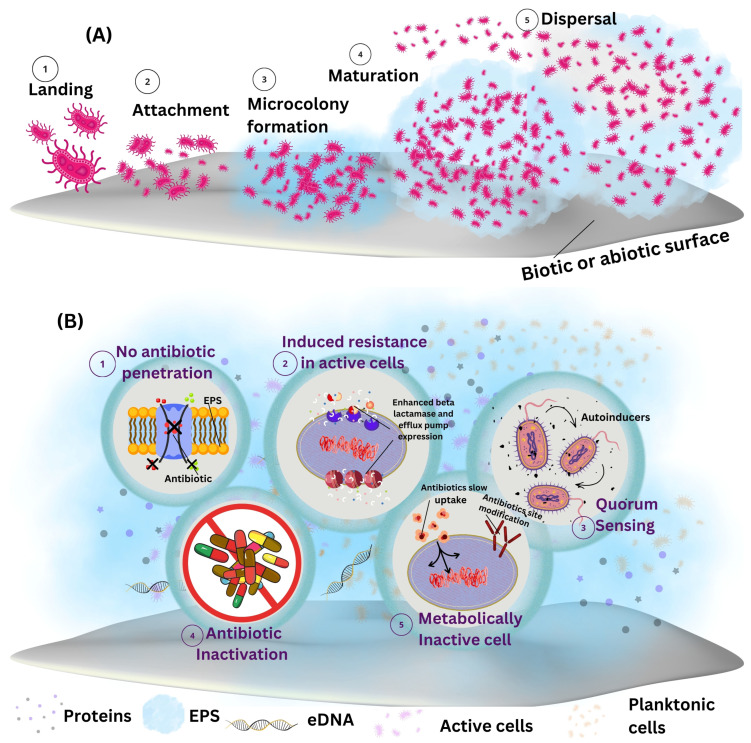
(**A**) Biofilm formation stages (landing to dispersal) (**B**) Key resistance mechanisms, including limited antibiotic penetration, quorum sensing, induced resistance, antibiotic inactivation, and persister cells that play an important role in AMR [13].

In veterinary medicine, knowledge of microbial biofilms and their key roles in disease progression is very limited compared to human medicine. The important role of biofilms in animal health is only now being acknowledged, and only a few studies have examined the clinical significance of microbial biofilms in veterinary medicine, which, in practice, leads to significant financial losses [1]. In recent studies, it has been observed that the microorganisms that trigger infections in hospital settings frequently mirror the same strains found circulating within veterinary medicine [2]. Their resistance is a major problem for public health, as they can be passed on to humans through the environment or by direct contact with infected animals [3,4]. Biofilms act as resilient reservoirs for chronic infections in the veterinary sector, fueling persistent and hard-to-treat conditions such as bovine mastitis, recurring urinary tract infections, and debilitating wound sepsis [5]. Besides catheters and wounds, the veterinary biofilms severely colonize surgical implants and dental surfaces, providing continuous reservoirs for chronic infection [6,7]. Furthermore, traditional antimicrobials fail in this situation because the protective matrix of a biofilm plays a major role in blocking antibiotic penetration and protecting the bacteria inside the biofilm by keeping them in a dormant state, as shown in Figure 1. This is one of the reasons that there is a need to find new, non-traditional therapeutic methods that can effectively overcome this resistance [8,9]. This need also arises because biofilms originate on biotic and abiotic surfaces, where physicochemical stability and interfacial cues drive microbial adhesion and initiate organized community development, leading to the failure of traditional antimicrobial agent action alone [10].

This problem was overcome by surface-based coatings that prevent biofilm formation by modifying the material interface to interfere with initial bacterial adhesion via controlled topography, wettability, and surface chemistry [11]. Antimicrobial surface coatings in the veterinary sector are one of the most effective ways to prevent infections in animals that arise from devices and the environment. These modified surfaces reduce the colonization of microorganisms at the origin, assist in minimizing antibiotic consumption, and help prevent the spread of resistant pathogens within animal health systems [12]. Through an integrative viewpoint on combination-based biomaterial coatings, this review focuses on a multifaceted approach to antimicrobial actions for a more effective biofilm prevention strategy. Existing reviews of antimicrobial coatings often highlight human biomedical applications, or they focus on one single coating material and completely disregard its translatability to veterinary medicine. This review gives a veterinary-based evaluation of combination-based biofunctional coating approaches from the aspect of animal health for orthopedic implants, wound management, catheterizations, dentistry and aquaculture. Veterinary findings are contrasted with human findings in this review, which also discusses the readiness of various coating technologies for translation to clinical settings and pinpoints their practical barriers. Also, it points out several critical research areas that currently hinder the use of coatings in clinic settings. This paper incorporates potential factors of antimicrobial resistance, biofilm prevention, and One Health concepts. Through this integrated approach, it aims to provide clear and practical guidance in developing the next generation of biofunctional coatings for veterinary medicine.

## 2. Literature Search Strategy

The literature included in this narrative review were identified through a structured search of the Google Scholar database to capture a broad range of peer-reviewed publications relevant to veterinary antimicrobial coatings and biofilm-associated infections. Searches were performed using combinations of keywords including veterinary antimicrobial coatings, biofunctional coatings, combination-based coatings, biofilm inhibition, orthopedic implants, catheter-associated infections, wound infections, dental implants, aquaculture biofilms, veterinary pathogens, antimicrobial resistance, nanoparticle coatings, polymer coatings, antimicrobial peptides, enzyme-based coatings, and quorum sensing inhibitors. Additional targeted searches were conducted for each major application discussed in this review to ensure the comprehensive coverage of veterinary-relevant coating technologies.

Priority was given to recent peer-reviewed research articles and review papers reporting antimicrobial and antibiofilm coating strategies applicable to veterinary medicine. Studies employing veterinary pathogens, animal infection models, veterinary medical devices, or veterinary clinical applications were preferentially included. Where direct veterinary evidence was limited, well-established studies from human medicine were incorporated only to illustrate technologies with potential translational relevance, and these are clearly identified as indirect evidence throughout the review.

Studies lacking sufficient experimental detail, duplicate publications, non-peer-reviewed reports, and articles unrelated to veterinary biofilm control or antimicrobial coating applications were excluded. Following title and abstract screening, potentially relevant studies were assessed for their scientific quality, relevance to the review objectives, mechanistic insights, and translational significance. This structured approach enabled the inclusion of current evidence while maintaining a clear focus on veterinary applications, translational potential, and future research directions.

## 3. Biofilm–AMR–Coatings Nexus: Targeting Resistance at the Surface Interface

**Figure 2:**
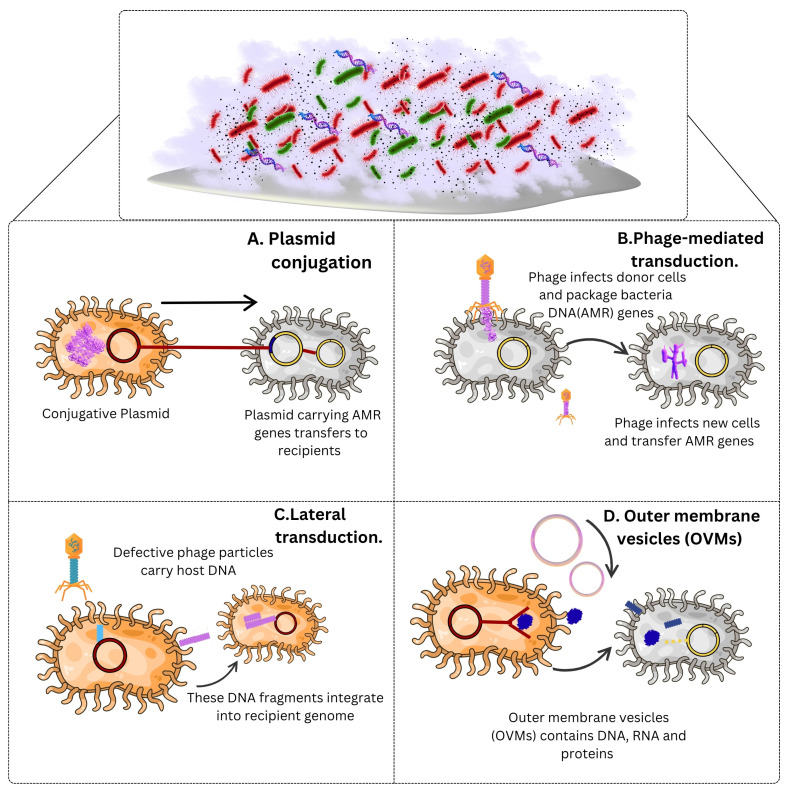
The horizontal gene transfer (HGT) routes in biofilms facilitate the dissemination of antimicrobial resistance genes among bacteria and provide bacteria with an advantage in their survival within biofilm communities, illustrating the major HGT mechanisms, including (**A**) plasmid-mediated conjugation, (**B**) bacteriophage-mediated transduction, (**C**) lateral transduction, and (**D**) outer membrane vesicle (OMV)-mediated transfer of genetic material. Collectively, these mechanisms accelerate the horizontal dissemination of resistance determinants, promote the emergence of multidrug-resistant bacterial populations, and contribute to the persistence of chronic biofilm-associated infections [21].

Biofilms are the core driver of AMR, as biofilms change vulnerable planktonic bacteria into highly resilient groups through three processes which include matrix protection, metabolic slowdown and structural heterogeneity. The dense structure of the organisms improves horizontal gene transfer through plasmids, phages, lateral transduction and outer membrane vesicles’ OMVs, making them active reservoirs of resistance, as illustrated in Figure 2. The internal gradients of the system produce two outcomes because they create different physical traits while enabling the survival of bacteria, which then develop resistance to antimicrobial treatment [14]. Biofilm-embedded bacteria receive protection from their EPS matrix, which contains eDNA, polysaccharides, proteins, and amyloids, because this matrix prevents antibiotic penetration while facilitating gene transfer and enabling bacteria to endure through quorum sensing and stress adaptation. The structured environment of this system facilitates the development of antimicrobial resistance, immune evasion, and ongoing infections, which demonstrate the necessity for specific anti-biofilm treatment methods [15,16]. Recent insights show that conventional antibiotics fail not only due to genetic resistance but because biofilms contain bacteria that enter dormant states, which makes them immune to drugs that target active bacteria. The combination of biofilm dormancy and biofilm structure enables bacteria to survive medical treatment, which leads to persistent recurring infections [17]. Antimicrobial coatings provide preventive benefits because they function at surface level, which serves as the first point where microbes attach to surfaces. The surface material prevents microbial attachment, which stops their initial biofilm creation and bacterial colonization until they have established their resistance mechanisms. The coatings protect against infection because they operate as a pre-emptive barrier that decreases selective pressure and antibiotic dependence to control the development and dissemination of antimicrobial resistance [18]. Interface coatings function as multiple operating systems that use their anti-adhesive surfaces together with their antimicrobial release, photodynamic effects, and phytochemical bioactivity to stop bacteria from sticking to surfaces while stopping biofilm development. The mechanisms operate as microbial elimination methods, which accomplish this through four different methods: they destroy microbial membranes, create reactive oxygen species, stop enzyme functions and block quorum sensing [19,20].

## 4. Veterinary Biofilms and Risk Factors

### 4.1. Major Veterinary Pathogens

Biofilms in veterinary settings exist as structured communities of microorganisms that attach themselves to animal tissues and medical devices while using their self-generated protective matrix. The biofilms create increased resistance against both antibiotics and host defenses, which results in persistent and challenging-to-treat infections in animals [6]. *Staphylococcus aureus* exists as a common pathogen in livestock that causes infections that lead to mastitis, dermatitis and joint infections. The bacteria use biofilm formation to establish persistent infections through their ability to strongly adhere to infected tissues or surfaces and animal-derived products [22]. Biofilm-forming *Escherichia coli* can cause mastitis by adhering to mammary tissues. The pathogen maintains its presence in the body, which results in extended illness duration, decreased treatment success rates, and higher negative effects on dairy production and animal well-being [23]. The hospital-associated pathogen *Pseudomonas aeruginosa* exists as a persistent organism that infects equipment, surfaces and water systems in veterinary clinical settings. This organism shows the ability to bond with non-living surfaces while creating protective biofilms which consist of polysaccharides, proteins and extracellular DNA. The infections that occur in clinical settings normally develop as opportunistic infections which begin in three types of body sites: open wounds, surgical wounds and areas of weakened immune function in animals [24]. On the other hand, *Salmonella* spp. create biofilms that enable the bacteria to survive when eggshell surfaces undergo cleaning and storage procedures. The contamination risk increases because this process establishes a pathway through which pathogens can transmit via eggs, which creates various food safety problems [25].

### 4.2. Environmental and Host-Specific Factors Influencing Veterinary Biofilms

Biofilms in veterinary settings enable pathogens to persist on animals, equipment, and food products, which results in increased resistance against antimicrobials and standard cleaning procedures. The ability to persist in various environments enables disease transmission between animals, humans and their surroundings, which creates One Health problems through the spread of zoonotic diseases and antimicrobial resistance [24,26]. Milk residues and moisture together with improper cleaning methods create conditions that allow microorganisms to establish themselves and multiply on the surfaces of milking equipment. The combination of surface roughness and temperature changes together with equipment operation establishes conditions that enable biofilm development and maintenance on surfaces [27,28]. Recently, it was demonstrated that the moisture on equine equipment and surfaces creates a favorable environment that supports microbial attachment, growth, and matrix formation. Biofilms establish themselves in wet environments because the moisture content enables them to survive for extended periods, which heightens the possibility of horse infections that affect wounds and hooves and common equipment [29,30]. Another important factor is polymicrobial biofilms in poultry that enhance pathogen survival by enabling cooperation among bacteria, increasing resistance to treatment [31].

## 5. Antimicrobial Coatings in Veterinary Medicine: Current Status

Presently, it is acknowledged that, just like humans, animals also require appropriate and thorough medical attention and treatment. A significant part of the healing process is coating surfaces with bioactive, biostatic, or antimicrobial compounds. These coatings can promote healing by reducing inflammation caused by microorganisms, making veterinary surfaces more effective [32].

### 5.1. Controlled-Release Systems

One of these coating classes is surface-controlled release coating, in which the antimicrobial action is governed by the surface area of the nanoparticles or other biomaterials embedded in the hybrid layer. This surface-controlled release coating provides active protection for veterinary implants, such as tibial plateau leveling osteotomy (TPLO), by releasing antimicrobial ions that disrupt bacterial cell membranes and prevent the formation of harmful pathogenic biofilms. The system effectively promotes animal bone healing and tissue regeneration, but relies on a delicate “safety window” due to two factors, which include host-cell toxicity and the loss of protective power from ion depletion and nanoparticle clumping (agglomeration) in biological fluids [33].

### 5.2. Anti-Adhesive Coatings

Similarly, anti-adhesive coatings are considered protective for veterinary food products, owing to their ability to reduce surface tension and prevent pathogen attachment and biofilm formation on products such as table eggs. These coatings also protect public health by preventing zoonotic disease transmission and reducing economic losses, as it stops *Salmonella* and *E. coli* contamination while extending food storage periods without causing chemical toxicity. The system uses a non-leaching anti-adhesive barrier as its main mechanism of action, although its active components have limited effectiveness against resistant *S. aureus* bacterial strains [34].

### 5.3. Contact-Killing Coatings

Contact-killing coatings provide a sustainable solution that combines their durable nature with their specific antimicrobial performance, which serves as an alternative to traditional disinfectants [35]. These coatings are effective in eliminating microbial contamination, as these contact-killing coatings offer a powerful strategy to combat microbial contamination by directly eliminating pathogens upon surface contact without releasing biocidal agents into the environment. These surfaces reduce biofilm formation, limit the spread of antimicrobial resistance, and provide long-term protection in healthcare settings [36]. Solid antimicrobial metal surfaces composed of high-purity electrolytic copper (99.9% Cu, C11000) and brass (70% Cu, C26000) have been designed for direct installation in poultry against biofilm formation by *Salmonella enteritidis*. Their antimicrobial performance is based on the inherent contact-killing ability of copper alloys, where direct bacterial contact triggers copper ion release, leading to membrane disruption and cell death. However, when copper was coated with tin to comply with regulatory standards, the coating prevented ion release and consequently abolished its antimicrobial effectiveness [37].

These limitations lead to the use of combination-based strategies that combine two or more agents to overcome challenges that individual bioactive compounds cannot (Table 1). This method achieves better results because it treats multiple microbial pathways simultaneously, leading to improved performance and reduced resistance development compared with single-treatment methods [38].

## 6. Combination-Based Biomaterial Coatings

Combination-based refers to when two or more agents join forces to produce an effect that exceeds their individual capabilities. The agents work together to enhance their respective abilities instead of functioning as separate entities. These antimicrobial systems demonstrate better performance because they require lower doses to achieve results that surpass the effectiveness of using a single agent [52,53]. Combination at the biomaterial–microbe interface refers to the cooperative interaction between engineered material surfaces and antimicrobial agents that together amplify their effect against microbes. This combined work of both components produces stronger bacterial inhibition and biofilm disruption effects than their individual capabilities [54,55]. In biomedicine, there are three major types of synergism.

### 6.1. Physical Combination-Based Approaches

Physical combination takes place when material or treatment actions achieve a level of combined physical interaction (e.g., surface charge, size, form, heat, light, mechanical force) that is greater than the individual interactions, resulting in the elimination of pathogenic microorganisms [56,57]. Physical combination plays an important role in the design of antimicrobial implants since it allows for the killing of bacteria through combined physical mechanisms; e.g., TiO_2_ nanorod arrays display enhanced antibacterial activity under irradiated 808 nm NIR that can kill single species biofilms through direct physical damage to bacterial cells [58]. Likewise, it was also recently observed that superhydrophilic coatings composed of layered nanoparticles along with antibiotics produced negative surface charge; the nano-/microstructure of its surface and hydrated layer formed a barrier that prevented the entrance bacteria and protein through electrostatic and steric repulsion, thus inhibiting the interaction of bacteria along with increasing the activity of the antibiotics embedded [59]. Graphene nanospike-made coatings, with their sharp edges and high defect density, create physical barriers that provide protection. The micro- and nanostructural elements of this system create mechanical disturbances that disrupt bacterial membranes while preventing biofilm formation and decreasing bacterial growth, showing the physical combined effect because the nanospikes shape their chemical oxidative stress properties to create an antibacterial effect that exceeds their individual effects [60].

### 6.2. Chemical Combination-Based Approaches

Chemical combination occurs when two or more chemical substances interact to produce an effect that exceeds their expected individual performance, as their mechanisms of action support each other during combined activity [61]. Antimicrobial mechanisms include reactive oxygen species (ROS) generation, which are used in both biomedicine and killing pathogens. The hyperaccumulation of superoxide radicals, hydroxyl radicals, and hydrogen peroxide damages DNA, proteins and lipids by attacking the cell membrane, leading to cell lysis. Multifunctional systems generate ROS in excess to increase killing and break resistance [62,63]. An example includes the three-metal Cu/Ag/Zn trimetallic coating on urinary catheters, which showed enhanced antimicrobial properties owing to the trio of metals. The involvement of the three metals led to increased bacterial killing: individual metals only release one toxic metal ion, while the combination of metals released highly reactive oxygen species. This enhanced generation directly led to the oxidative destruction of proteins, lipids and DNA of bacterial biofilms [64]. Ion release is another potential scenario during chemical combination: for example, in medical surfaces designed to inhibit infection. A coordination-based antibiofilm coating made from tannic acid was used on these surfaces, which coordinated with Cu^2+^ and Ga^3+^ ions. The coating exploited the acidic biofilm to release Cu^2+^ onto the membranes of bacteria, damaging them using oxidative stress and a Trojan horse mechanism that released Ga^3+^ into the bacteria, which inhibits iron metabolism [65]. In a comparable fashion, the Ag/TiOx composite layer triggered by visible light exhibits higher photocatalytic effectiveness towards well-formed biofilms owing to the combined benefits of plasmonic silver and defect-controlled TiOx. The coupling of Ag with TiOx increases charge separation as well as the generation of reactive oxygen species, resulting in efficient biofilm eradication under harmless visible light irradiation. The incorporation of PDMS in the layer composition improves the durability of the layer, making it possible for the layer to have antibacterial activity after repeated use without antibiotics [66].

### 6.3. Biological Combination-Based Strategies

Biological combination is a dynamic interaction where multiple biological agents or systems coordinate their functions to amplify a shared outcome beyond their independent capacities [67]. Recently, biological combination was used to combat the problem of the high risk of infection and biofilm build-up during the long-term use of Schanz pins due to their common use in both medicine and veterinary practices. The combination of Vancomycin and Gentamicin within a PLGA nanosphere delivery system on these pins would have a combination effect. They would act on the bacteria by simultaneously preventing cell wall synthesis and protein production. This attack would kill the bacteria, preventing biofilms from forming on the implant. The material is degraded in a controlled fashion, enabling the drugs to stay on the implant for more than 40 days to kill the bacteria that would survive a single short-term treatment [68]. On top of that, it is possible to achieve a biological synergism from a p(HEMA) copolymer matrix with two antibiotic drugs, Ampicillin (AMP) and Levofloxacin (LVX). This comes from their synergy since Ampicillin is an irreversible transpeptidase inhibitor, inhibiting cell wall synthesis, and Levofloxacin is a DNA Gyrase inhibitor, inhibiting bacterial replication. In addition, their antimicrobial effect is maintained for 14 days due to them being able to co-function by inhibiting two vital cell processes as well as repelling negatively charged bacteria from forming mixed-species biofilms. However, reduced structural integrity (tensile strength) when loading higher concentrations of ampicillin into p(HEMA) hydrogel limits its prolonged action against pathogens [69]. This limitation was overcome by the incorporation of AMP-mediated coatings that showed a prolonged antibiofilm effect by a “repel-and-kill” mechanism. While GL13K antimicrobial peptides provide the active killing power by disrupting bacterial membranes, elastin-like recombinamers (ELRs) provide a passive, low-fouling shield that prevents bacteria from even reaching the surface. Furthermore, these AMP-mediated coatings stay active longer by preventing the survival of heterogeneous bacterial populations that typically lead to medical device failure [70]. Comparable to poultry, the pig industry struggles with ongoing infections because biofilms protect harmful bacteria on farms and in pig wounds. This makes infection control harder, raises production costs, and contributes to antibiotic resistance [71]. For this purpose, [72] made a PVA-based silver gelling fiber dressing that demonstrated strong antibiofilm activity in a porcine wound model through its ability to destroy MRSA biofilms and its ongoing silver ion release, which reduced bacterial contamination. The product used its hydrophilic design to create a wet wound environment that established better wound healing conditions while preventing bacteria from multiplying.

## 7. Fabrication Strategies for Bioactive Coatings

**Figure 3:**
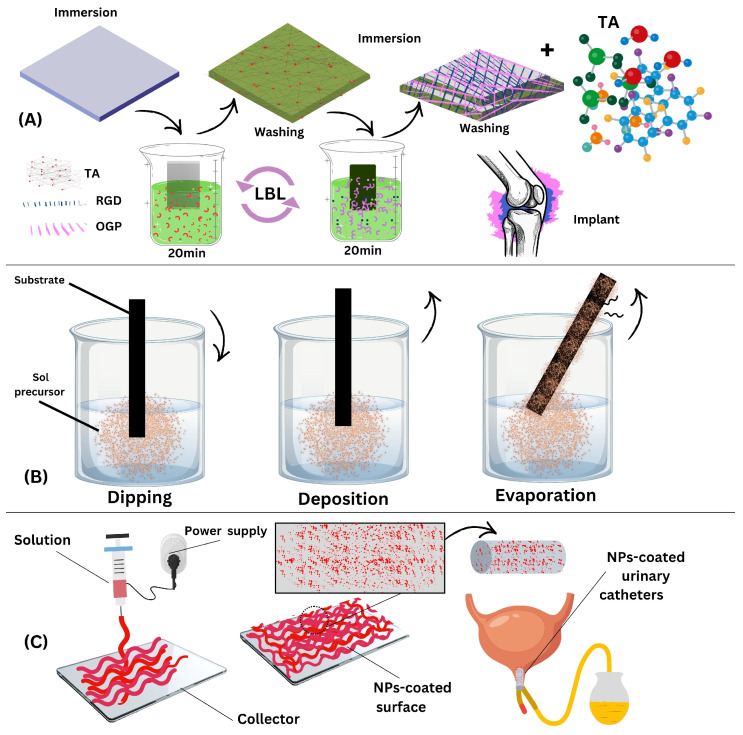
Schematic of coating fabrication methods: (**A**) layer-by-layer assembly, (**B**) dip-coating, and (**C**) electrospinning, used to create functional anti-biofilm surfaces on implants and catheters.

There are a variety of deposition techniques used to create functional coatings which enhance biological and physicochemical performance for surface material modifications. The methods of dip-coating, electrospinning and layer-by-layer assembly, as depicted in Figure 3, enable precise control over the production of thin films that exhibit both antimicrobial and antibiofilm characteristics. The methods have become popular because researchers want to make their coatings more durable and effective, which applies to both biomedical and veterinary fields [73]. On the other hand, the deposition method of dip-coating involves dipping a substrate into a coating solution, which creates a thin film that dries when the substrate is lifted at a specific speed. This method is commonly used in biomedical applications to apply coatings on catheters and implants because the resulting layer enables the release of antibiotics and polymers and nanoparticles, which work to prevent bacterial growth and biofilm formation [74,75]. The layer-by-layer (LBL) process is a method of depositing thin films whereby surface generation can be achieved through the stepwise adsorption of oppositely charged materials. This technology has been applied in biomedical fields as a coating strategy for implants and catheters that is capable of delivering antimicrobial peptides, antibiotics, polymers, and nanoparticles simultaneously [76,77]. The spray-coating method is one way of depositing material by spraying a solution containing the coating material on a surface, forming a thin film. The technique has been utilized to coat catheters and implants quickly [78,79]. The sol-gel method establishes a coating process which transforms liquid precursors into a gel that creates a permanent thin film that adheres to surfaces. The process of embedding antimicrobial drugs into the coating matrix enables controlled drug release, which effectively prevents bacterial growth and biofilm development [80,81].

## 8. Functional Classes of Combination-Based Biomaterial Coatings

Scientists have developed multiple nanocoating combinations that successfully destroy harmful microorganisms through their combined usage. These advanced surfaces integrate multiple antimicrobial mechanisms to enhance biofilm disruption and prevent microbial persistence [82]. There are numerous technologies that enable coatings to create biomimetic layers with high surface areas that can store and release antimicrobial peptides, antibiotics and nanoparticles to maintain their antimicrobial and anti-biofilm properties [83,84,85].

### 8.1. Anti-Adhesion and Bactericidal Combination Systems

These systems are especially developed to both stop microbial attachment and destroy cells that have already been attached. These coatings achieve their biofilm protection through the combination of surface-repelling properties and different killing mechanisms [86]. Hydrophilic components in antimicrobial polymers provide researchers with a method to achieve precise control over hydrophobic and hydrophilic properties, which directly impacts bactericidal effectiveness and safety of the materials. The groups establish their antibacterial capabilities through their power to control polymer solubility and membrane binding and biocompatibility, which results in improved antibiofilm protection without increasing toxic effects [87]. Avian Pathogenic *Escherichia coli* (APEC) serves as the main bacterial pathogen that causes colibacillosis in poultry according to veterinary medicine, resulting in substantial economic losses for nations worldwide [88]. The polymer coating on ZnO nanoparticles exists because PEG-6000 creates a protective layer which works together with antibiotics. The hydrophilic chains of the material form hydrogen bonds and electrostatic bonds to the surface of the nanoparticle. The coating maintains particle stability by preventing clumping, which results in improved particle distribution and better contact with APEC cells. The process produces more Zn^2+^ ions together with increased ROS generation. The increase in membrane damage leads to stronger antimicrobial effects and combined effects of ZnO-NPs against multidrug-resistant APEC strains [89]. Recently, scientists discovered that microbial biofilms that develop on eggshells create a protective shield that enables bacteria to endure extreme environmental conditions while maintaining their biological functions for extended periods. These biofilms protect pathogens from cleaning agents, which raises the risk of contamination and makes it easier for diseases to spread through food and poultry [90]. This problem was solved by phage-loaded hydrophilic polymer film coatings for antibacterial food protection, which work by inhibiting biofilm development through its active phages (PBSE191) that target and destroy *Salmonella enteritidis*, resulting in reduced bacterial contamination on eggshells. The process prevents biofilm development by blocking initial attachment and microcolony formation, while the hydrophilic PVA matrix ensures continuous phage release and preserves phage stability [91].

### 8.2. EPS-Disrupting and Microbial-Killing Coatings

Biofilm development starts when bacterial cells are surrounded by extracellular polymeric substances (EPS), which form a protective matrix. The biofilm develops its complex three-dimensional structure as the biofilm progresses through its growth cycle. The EPS matrix structure enables bacterial cells to interact with one another while protecting them from harsh environmental conditions. Biofilms demonstrate increased resistance to antibiotics because of this particular property [92]. A highly potent coating formed by DNase I and cellobiose co-immobilization on the surface of positively charged chitosan nanoparticles was created to form a dual-function antimicrobial delivery system. DNase I acts as a molecular “scaffold-breaker” by breaking down extracellular DNA, which helps to dissolve the biofilm’s structural glue. As the matrix becomes thinner, CDH can move deeper into the biofilm, where it produces antimicrobial agents that target and neutralize the pathogens inside [93]. Similarly, the use of peptide-based hydrogels synergistically with nanoparticles, such as silver or gold nanoparticles, also acts as a stabilizer and delivery vehicle in numerous infections. The coating uses stabilized nanoparticles to get through the dense polymicrobial matrix and produce reactive oxygen species (ROS). This process breaks down the biofilm’s structure and disrupts the EPS [94]. In the veterinary sector, a hydrophilic anti-adhesive polymer layer was first applied on polyurethane catheter surfaces in a mouse urinary infection model to reduce bacterial attachment by making the surface less favorable for colonization. On top of this layer, a surface-tethered antimicrobial peptide (AMP)—such as the cysteine-modified peptide RRWRIVVIRVRRC—was chemically immobilized. This created a dual-function coating where the polymer acts as a physical anti-fouling barrier, while the AMP provides active antimicrobial killing by the deeper disruption of bacterial membranes [95].

### 8.3. Quorum-Sensing Interference-Based Combination Coatings

Quorum sensing is used by animal pathogens for regulating communication between bacteria, resulting in the synchronized production of virulence factors and biofilms, causing a more serious infection. By interfering with bacterial communication using quorum sensing interference, infections can be controlled because pathogenicity will be reduced and antimicrobial therapy will be more effective [96]. In recent times, carbon nanoparticles (candle soot), poly(HEMA-co-APBA) polymer, and QS inhibitors, including quercetin and baicalein, have been combined in order to interfere with bacterial communication, since enzymes like acylase degrade the signaling molecules such as AHLs and thereby inhibit bacterial coordination for biofilm formation [97]. A dual-function antibiofilm coating was developed by processing candle soot carbon nanoparticles to develop a coating that combined photothermal properties with nitric oxide (NO) release through S-nitrosothiol functionalization. The system generates focused heating through near-infrared light, which activates fast NO release to kill bacteria, while continuous NO release without light exposure stops biofilm development. The research demonstrated strong antibacterial effects and effective biofilm prevention together with good compatibility testing through both laboratory experiments and rat model studies [98].

## 9. Veterinary-Specific Applications of Combination-Based Coatings

Microbial colonization constitutes a significant problem within veterinary medicine because it affects both implanted medical devices and coated surfaces that veterinarians use on their animal patients (Table 2). Bacteria can establish adhesion to surfaces within a few weeks after their placement, and they proceed to multiply and develop biofilms which exhibit increased growth rates as time progresses. The infections cause two main problems because they create inflammation and degrade device functionality, which causes harm to animal health, leading to the need for the development of effective antimicrobial coating methods for use in veterinary medicine [12,99].

### 9.1. Orthopedic and Dental Implants

**Figure 4:**
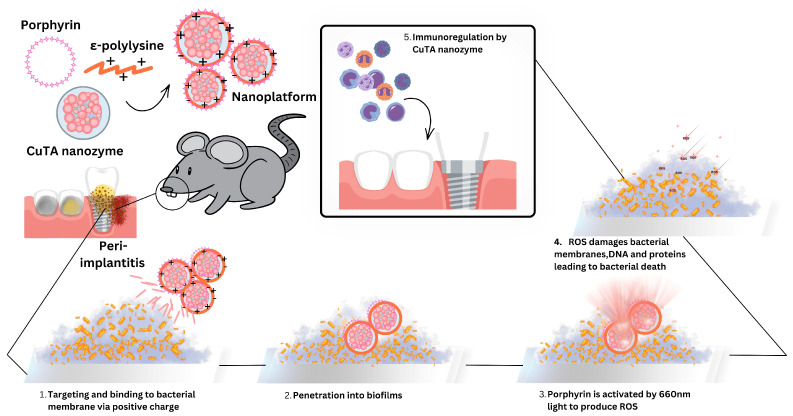
Combined nanocoating mechanism where a porphyrin–ε-polylysine–CuTA platform targets bacteria, penetrates biofilms, and generates ROS under light activation, leading to bacterial killing and immune modulation in rat model.

Biomaterials form the essential foundation of modern orthopedic procedures and implant development. Orthopedic devices cause multiple functional issues which include implant instability that occurs without infection, problems of insufficient bone integration, microbial biofilm development and postoperative infections in the veterinary sector [110]. In a direct way, a biofilm, or infection, shows inflammation to the dental implant surfaces, causing the periodontal tissue to reside that results in bone loss [111]. An antibiofilm coating which protects orthopedic titanium implants from bacterial biofilm growth and implant-related infections by utilizing the 5-(4-bromophenyl)-N-cyclopentyl-1-octyl-1H-imidazol-2-amine compound was fabricated. This coating worked better when combined with the antibiotic cefuroxime, as the two together reduced biofilm cells more effectively than the antibiotic alone. The coated implants demonstrated effective antibiofilm protection in rabbit osteotomy model tests while permitting bone healing and integration [108]. Furthermore, an in vivo model with a femoral implant infection in mice was established to examine the effectiveness of combined “cocktail” treatment with gentamicin, curcumin and isobavachalcone. These components are enhanced for the antibiofilm and antibacterial activity of inhibiting osteolysis. It revealed that the synergism of “cocktail” therapy in killing MRSA biofilm is excellent, which could contribute to killing MRSA via attacking bacterial membranes, inhibiting protein synthesis and disturbing resistance-related pathways compared to the monotherapy [112]. Peri-implantitis is an inflammatory condition that develops around a dental implant after its surgical placement. The main reason for this condition to develop arises from bacteria that form biofilms on the surface of the implant [113]. Recently, a combined nanoplatform to treat peri-implantitis using a rat model was made, as shown in Figure 4. The system uses photodynamic therapy with porphyrin and 660 nm laser light to create ROS that destroy biofilms. This system also uses ε-polylysine to enhance its ability to target and penetrate bacterial cells. A CuTA nanozyme protects cells from oxidative damage by removing excess reactive oxygen species while it helps regulate immune response through macrophage activation [114].

Other than nanoparticles, peptide-based coating has also shown great potential in osseointegration in vitro and in vivo. The Ag–GL nanocomposite coating uses silver nanoparticles and the antimicrobial peptide GL13K to create a system with combined antibiofilm protection. It achieves two antibacterial effects: silver ion release and GL13K contact-based membrane destruction. The coating reduced infection rates and improved bone growth and implant integration during mechanical testing in the rat implant model [115]. Another hybrid coating made from silver, zinc oxide, and copper nanoparticles works to stop bacteria from sticking to surfaces and forming biofilms through its combined antibacterial properties. The system achieves its antibacterial effect through three mechanisms which include ion release, oxidative stress creation and bacterial membrane disruption. Antimicrobial coatings play a critical role in veterinary medicine by protecting TPLO implants used in canine orthopedic surgery, as this system helps reduce infection risks after surgery while it stops biofilm formation on implants, and it enables the rapid recovery of bone and soft tissue, which allows animals to heal faster [33].

### 9.2. Wound Dressings and Surgical Materials

**Figure 5:**
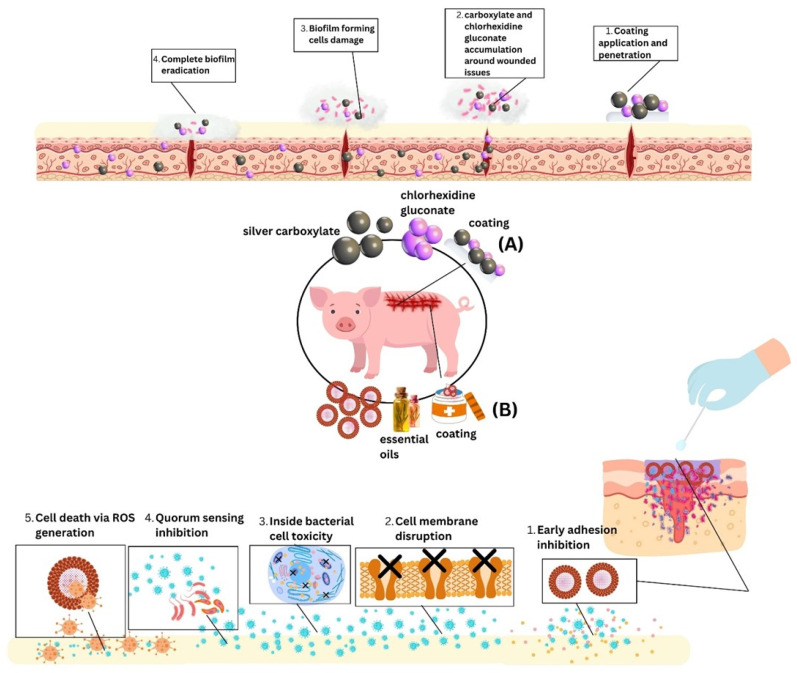
Schematic diagram showing two-component coating system studied using porcine wound model system. (**A**) A combination of silver carboxylate and chlorhexidine gluconate provides tissue penetration, local concentration, and progressive destruction and eventual elimination of biofilm. (**B**) An alternative method utilizing natural essential oil-based compounds along with NPs prevents early attachment of bacteria, disrupts cell membrane structure, creates intracellular toxicity, inhibits quorum sensing, and facilitates ROS-mediated bacterial killing.

Biofilms in wounds and surgical sites are organized groups of microbes that attach firmly to human tissue and medical devices. They make infections hard to treat and often lead to long-lasting or repeated problems [116]. The use of hybrid or combination-based coatings provides a method to achieve constant antibacterial protection while operating at predetermined time intervals, as presented in Figure 5. The solution shows its effectiveness by providing protection against infections that occur during the initial stage of inflammation and the subsequent process of wound and surgical site healing [117]. A nanobiomaterial-based antimicrobial formulation with biosynthesized silver nanoparticles conjugated with plant oils works as a bioactive coating solution against pathogens present on the skin of animals. This nano-formulated ointment helps in wound treatment by delivering its antibacterial power through silver nanoparticles that destroy bacterial membranes and induce oxidative damage while the plant-based phytochemicals stop bacterial attachment and control quorum sensing and EPS production, which leads to combined effects that block and disrupt wound-related biofilms together with improved tissue recovery time [118]. *Cutibacterium acnes* and MRSA create strong biofilms which enable them to survive on body tissues and medical implants because these biofilms protect them from both immune defense mechanisms and antibacterial treatments [119,120]. Researchers tested an antimicrobial coating made from silver carboxylate and chlorhexidine gluconate on Yucatan pig skin to see if it could pass through skin barriers and kill different bacteria. The coating showed ongoing antibacterial effects at the skin–device boundary, effectively controlling harmful bacteria like MRSA and *Cutibacterium acnes* during both early bacterial attachment and later biofilm growth [121]. Furthermore, the core–shell electrospun nanofibers showed a cooperative antibiofilm ability by combining two effects: minocycline-based bacterial protein synthesis inhibition and *Gymnema sylvestre* phytochemicals that disrupt bacterial biofilm adhesion, quorum sensing, and extracellular polymeric substance formation. Together, these effects prevented biofilm development on burn wound surfaces in the porcine model. Reduced microbial colonization leads to better re-epithelialization and improved collagen organization, resulting in faster wound healing [122].

### 9.3. Catheters

**Figure 6:**
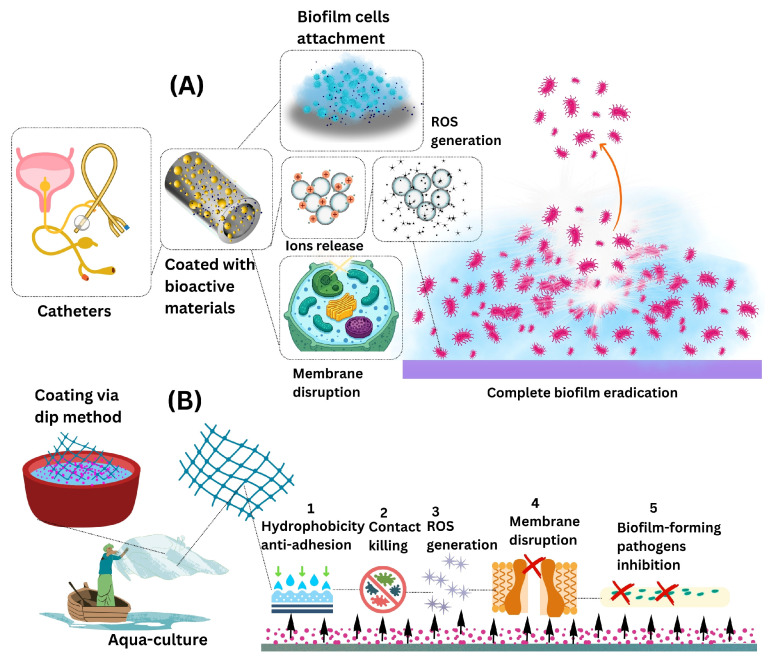
Schematic of combined antimicrobial coatings for medical and aquaculture use. (**A**) Bioactive-coated catheters release ions, generate ROS, and disrupt bacterial membranes to eliminate biofilms. (**B**) Dip-coated aquaculture nets exhibit anti-adhesion, contact-killing, ROS production, and membrane damage, preventing biofilm formation.

Catheter-associated infections emerge when microorganisms establish contact with an indwelling catheter surface and subsequently form biofilms, which protect the microorganisms from being eliminated. These infections occur frequently in medical environments because they result from the extended use of catheters, which increases both patient suffering and healthcare system demands [123]. Combined coatings play a crucial role in catheterization using a silicone urinary catheter in a rabbit model (Figure 6A). Silver-and zinc-based combination-based coatings performed well in vivo in terms of both antibacterial and antibiofilm effects through decreased bacterial colonization onto the catheter and suppressed inflammation of the adjacent tissues. An internal layer coating of Ag nanoparticles killed bacteria immediately while the outer porous zinc coating controlled silver release and produced ROS to kill bacteria enduringly [124]. Peripherally inserted central catheters (PICCs) are prone to microbial adhesion, which results in biofilm development and increased central line-associated bloodstream infection (CLABSI), the most serious catheter complication. Hydrophobic polymer-based technology PICC biomaterials are available in an attempt to resist microbial adherence and biofilm development [125]. One such biomaterial hybrid coating was applied to peripherally inserted central venous catheters (PICCs) using zwitterionic and fluorinated materials to create a dual super-hydrophilic and super-hydrophobic surface. This design effectively reduced protein absorption, platelet adhesion, and biofilm formation by *Escherichia coli* and *Staphylococcus aureus*. Tests in rabbit and pig models showed reduced thrombosis and effective antifouling performance in living conditions [126]. Another multifunctional coating was developed for interventional cardiovascular catheters. Researchers tested it in a canine jugular vein model, demonstrating its ability to reduce thrombosis and microbial colonization. The coating combined heparin sodium and ethyl lauroyl arginate to create a surface system with both anticoagulant and cationic antimicrobial properties. Heparin blocked fibrinogen adsorption and cell adhesion by stopping the formation of biofilms. Ethyl lauroyl arginate destroyed bacterial membranes, resulting in an over 99% reduction of *Escherichia coli* and *Staphylococcus aureus* and preventing biofilm development at all stages [127].

### 9.4. Aquaculture Systems

Aquaculture biofilms function as natural habitats for pathogenic microorganisms that include *Vibrio*, *Aeromonas* and *Pseudomonas* and multiple fungal species that establish protective communities through their attachment to submerged surfaces. The structured microbial layers provide enhanced protection against both environmental stressors and antimicrobial treatments. Biofilm-associated pathogens create permanent infection reservoirs that lead to increased disease outbreaks and financial damage within aquaculture systems [128,129]. In recent years, a combination polyurethane–methylphenyl silicone resin and polyhexamethylene guanidine hydrochloride (PHMG) coating has been developed to prevent biofilm infections through two mechanisms: decreasing microbial attachment and destroying surface bacteria. The water-repellent surface with its minimum energy properties prevents objects from sticking, while PHMG destroys cellular membranes. This method decreases pathogen accumulation on aquaculture nets (Figure 6B), resulting in lower disease transmission [130]. In addition to this, a second coating was created by embedding a nanocomposite (ZOPPMGO and ZSPPMGO) within a polydimethylsiloxane matrix (PDMS) that was then sprayed with a further nanocomposite layer to ensure continued activity. This coating acts as an effective barrier to biofilm growth through three methods: reducing surface energy, developing enhanced hydrophobicity and forming micro/nano roughness which repels microbes. The nanocomposites produce antimicrobial properties together with reactive oxygen species (ROS), which result in the death of bacteria that adhere to surfaces [131].

### 9.5. Mastitis-Associated Infections

Mastitis is an inflammatory condition that mainly affects the udder, and it is usually triggered by bacterial invasion. It is one of the leading diseases on dairy farms affecting both the quantity and quality of milk as well as the health and well-being of animals [132]. Most of the bacterial pathogens that can cause mastitis in the veterinary sector are *Staphylococcus aureus*, *Streptococcus agalactiae*, *Escherichia coli* and *Streptococcus uberis*. These microorganisms are capable of forming infections that persist for a long time by employing many virulence mechanisms like attachment to mammary epithelial cells, biofilm production, toxin generation, immune escape and more effective iron uptake. Overall, these aspects are responsible for tissue destruction, the slowing of bacterial elimination, and chronic intramammary infections [133,134]. To cop this infection, a antimicrobial teat coating primarily composed of zinc oxide nanoparticles (ZnO NPs) and lemongrass essential oil (LGEO) was incorporated into an adhesive polymeric sealant matrix. The polymer forms a stable protective film on the teat surface, while ZnO nanoparticles and LGEO provide sustained broad-spectrum antimicrobial activity. Together, these components create a durable barrier that prevents bacterial entry into the teat canal and helps reduce the risk of bovine mastitis during the dry period [135]. Recently, formulations incorporating gold, silver, copper, platinum, and iron-based nanoparticles into beeswax, oil, or glycerin–propylene glycol hydrocolloid matrices have been developed that adhere to the teat canal and create a protective coating. The nanoparticles eliminate pathogens by disrupting bacterial membranes, generating reactive oxygen species, and interfering with essential cellular functions, while the sealant acts as a physical barrier that blocks microbial entry. Although these formulations have demonstrated broad-spectrum antimicrobial activity with minimal cytotoxicity in laboratory studies, their efficacy remains largely confined to in vitro investigations, and issues related to long-term safety, formulation stability, regulatory approval, and validation against clinical field isolates must be addressed before widespread veterinary application [136,137].

## 10. Translational Challenges and Regulatory Considerations

Surface modification through coatings provides an effective solution to these problems. The application of functional coatings results in better treatment outcomes when compared to traditional drug therapies because these coatings deliver bioactive agents directly to targeted treatment areas [138]. The process of translating these coatings into clinical applications faces difficulties because they require proof of both their performance and their cost-effective implementation. Industrial adoption will remain impossible because expensive coatings with minimal extra advantages do not meet the essential requirements of production. The commercialization of advanced multifunctional coatings, which change manufacturing methods and shorten product shelf life, will face obstacles, making point-of-care applications the most suitable solution [139].

Veterinary medicine has received benefits from nanotechnology through its development of targeted drug delivery systems which produce superior vaccines and enable improved disease detection methods that result in more effective medical treatments. However, the main disadvantages of the system include its potential to cause toxic effects through oxidative stress, inflammation, tissue damage and its environmental threat, which comes from the buildup of nanoparticles in the environment. The system faces limitations because of insufficient regulations together with missing safety testing standards and unknown effects which might occur over extended periods in animal populations and natural environments [140]. Similarly, the use of AMPs in coatings faces limitations because they experience rapid enzymatic degradation, which leads to their unstable performance over time. Problems were also noted regarding controlled release functions and with the durability of the product under difficult long term usage conditions within veterinary medicine [141]. Another important issue is that many polymer-based therapies are developed for use in human medicine and subsequently tested or modified for application in animals, resulting in poor species-specific optimization, inefficient treatments and unpredictable animal behavior [142]. Currently, there is no integrated regulatory framework for evaluating antimicrobial coatings that is applicable to different industries including medical, marine and food. This situation results in inconsistent assessment of antimicrobial performance over time together with coating durability and their leaching characteristics. Regulatory agencies including the FDA, EMA, and EU Biocidal Products Regulation (BPR) now require manufacturers to provide substantial scientific proof that antimicrobial coatings deliver identifiable therapeutic benefits beyond standard uncoated products [143].

There are numerous practical limitations; mechanical stability remains a critical challenge for antimicrobial coatings used in veterinary medicine, as they must withstand surface wear, abrasion, friction during implantation, and continuous mechanical loading associated with animal movement and weight-bearing. Poor adhesion may lead to coating delamination or cracking, compromising antimicrobial performance and increasing the risk of implant-associated infections [144]. Sterilization can also alter the structural integrity and antimicrobial performance of surface coatings, particularly those containing heat-sensitive polymers or bioactive compounds [145]. Cytotoxicity comprehensive biocompatibility is also essential to ensure that antimicrobial coatings effectively prevent infections without adversely affecting surrounding tissues. Optimizing coating composition and dosage will be critical for achieving a balance between antimicrobial efficacy and host tissue safety [33].

## 11. Conclusions and Future Perspectives

**Figure 7:**
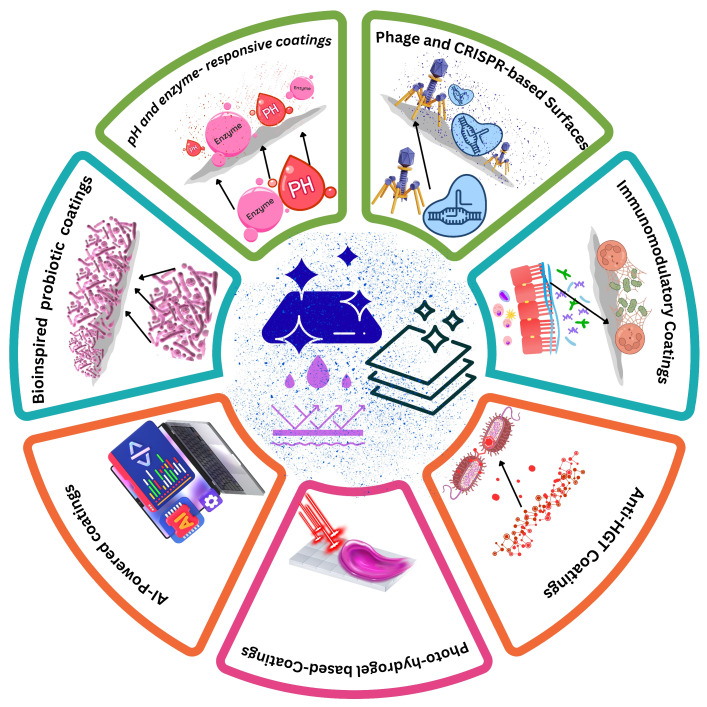
Future strategies for combined bioactive coatings, combining advanced materials and smart technologies to control biofilm-associated infections.

The development of bioactive antimicrobial coatings has made significant progress, yet their use in veterinary medicine remains restricted, which creates a significant gap in the One Health framework. Future developments will focus on smart coatings and stimuli-responsive coatings that use pH and enzyme systems to release antimicrobial agents, enabling controlled treatment delivery that minimizes harmful effects. Probiotic coatings along with bioinspired coatings using EPS-like compounds and antimicrobial peptides/bacteriocins also provide a more eco-friendly approach to inhibit biofilm development and bacterial resistance. In addition, AI can serve an important role in designing customized coatings for implants according to each species by predicting better surface chemistry, accelerating the biomaterial selection process, and optimizing osseointegration techniques in diverse animal models. Numerous biomaterials may actually work as strong alternative options to conventional veterinary implant surfaces, where phage-loaded coatings and CRISPR based surfaces can provide aimed antimicrobial activity against animal pathogens. Meanwhile, stimuli-responsive hydrogels and photothermal nanocoatings could behave like smart treatment platforms that can release bioactive agents on their own or inhibit bacterial population bacteria once they detect infection-specific physiological signals. Immunomodulatory and microbiome-preserving coatings may further be employed to suppress chronic inflammation and prevent dysbiosis, while anti-HGT coatings could serve as a frontline barrier against resistance gene dissemination in veterinary clinical settings as mentioned in Figure 7.

These strategies will push forward the goals of decreasing antibiotic use in food animals and therefore will contribute to combating the AMR threat to human and animal health systems. While AI is well suited for the rapid design of materials and for formulating, optimizing, and predicting antimicrobial activity, its use in veterinary coating studies is in its infancy. This is primarily due to the limited availability of standardized veterinary datasets, insufficient validation using veterinary-specific in vivo models, and the lack of integrated computational frameworks linking material properties with biological and clinical outcomes. Addressing these limitations through multidisciplinary collaboration and high-quality data generation will facilitate the broader adoption of AI-driven coating development in veterinary medicine. This field is developing smart antimicrobial interfaces which prevent infections, but more veterinary research still needs to occur before the field can implement these innovations to create One Health solutions.

## Figures and Tables

**Table 1:** Comparison of antimicrobial coating strategies in veterinary medicine and summary of the major antimicrobial coating strategies, their mechanisms of action, veterinary applications, and key functional limitations. It also highlights the principal challenges affecting their clinical translation, including stability, biocompatibility, antimicrobial durability, and scalability.

Coating Type	Common Materials	Advantages	Mechanism of Action	Key Limitations
Contact-killing, anti-adhesive, antibiofilm	Vancomycin; titanium implant surface (plates)	Strong antibiofilm, supports bone healing, localized action	Cell wall synthesis inhibition, prevents adhesion, biofilm inhibition	Resistance risk, limited spectrum (Gram+), surface-bound stability [39]
Active release, antibiofilm	CZ-01127, silicone (Si) polymer, titanium (porous-coated implants)	Localized protection, effective biofilm killing, supports bone ingrowth	Sustained antimicrobial release, biofilm eradication, planktonic killing	Incomplete joint-space eradication, diffusion limits, optimization needed [40]
Active release, antibiofilm	CZ-01127, silicone (Si), titanium surfaces	Strong MRSA killing, superior to vancomycin/gentamicin, effective early protection	Burst release, rapid bactericidal action; biofilm eradication	Short-term release; limited duration [41]
Anti-adhesive, antibiofilm	Sericin; titanium (Ti-6Al-4V), APTES, glutaraldehyde (GLU)	Biocompatible; reduces biofilm (~53%), simple modification	Inhibits bacterial adhesion, reduces biofilm formation	Moderate efficacy, limited spectrum [42]
Contact antimicrobial nanoparticle HA coating	Hydroxyapatite nanoparticles, silicone latex catheter	Reduces bacteriuria, decreases biofilm thickness, biocompatible, no urothelial toxicity observed	Silver ion release and surface contact-killing and inhibition of bacterial adhesion and biofilm formation	Limited long-term data, ion depletion [43]
Anti-adhesive	mPEG-DOPA3 polymer, catheter surface material	Reduces urinary bacterial load, lowers invasive infection rate, biocompatible, no tissue toxicity	Reduced bacterial attachment and inhibition of biofilm formation	Possible encrustation in some coatings, limited clinical validation [44]
Contact-killing	Silicone catheter, ZnO nanoparticles,	Strong antibiofilm effect, improved durability with protective layer, maintained activity in urine, improved safety	Surface contact-killing and ZnO ion release and enhanced antibiofilm activity with protective barrier layer	ZnO dissolution without protection, reduced long-term stability, regulatory constraints [45]
Drug release, hydrophilic coating	Nitrofurazone, Catheter surface materials	Strong antimicrobial durability, broad activity against multiple pathogens, effective biofilm reduction	Drug release and species-dependent antibacterial activity and biofilm inhibition	Species-dependent efficacy, possible resistance, variable performance across coatings [46]
Contact-killing	Titanium surface, Ag nanoparticles	Strong reduction in bacterial load, reduced biofilm formation, long-term nanoparticle retention, effective in vivo performance	Contact-killing via Ag^+^ release and inhibition of MRSA adhesion and biofilm formation on implant surface	Potential metal ion toxicity risk, long-term safety concerns [47]
Contact-killing	Gentian violet, PICC catheter material	Strong antibiofilm activity, effective against MDR bacteria and fungi, reduced thrombosis and inflammation, biocompatible in vivo	Broad-spectrum contact-killing and inhibition of bacterial and fungal adhesion and biofilm formation	Limited long-term clinical data, trace systemic CHX exposure, short animal study duration [48]
Contact-killing	Titanium plate, Mel4 antimicrobial peptide	Effective against *S. aureus* and *P. aeruginosa*, reduces inflammatory response (IL-1, TNF-α), supports infection control in vivo	Peptide-based contact-killing and inhibition of bacterial adhesion and biofilm formation with anti-inflammatory modulation	Limited clinical translation, peptide stability concerns, short-term animal study [49]
Anti-adhesion	Titanium plate, caerin 1.9 peptide	Reduces bacterial load, lowers inflammatory response, improves wound healing indicators, effective in mixed oral infection model	Peptide-based antimicrobial action with inhibition of bacterial growth, adhesion, and biofilm formation and modulation of inflammatory response	Peptide stability issues, short-term animal study [50]
Controlled release coating	OP-145 antimicrobial peptide, Polymer-Lipid Encapsulation Matrix (PLEX), implant surface	Sustained antimicrobial activity, high reduction in implant infection, effective bone and soft tissue sterilization in vivo	Controlled zero-order peptide release with initial burst and inhibition of *S. aureus* adhesion and biofilm formation	Initial burst release, partial infection persistence, limited long-term clinical validation [51]

**Table 2:** Comparison of combination-based biofunctional coating strategies and their biological effects across different veterinary animal models.

Combination-Based Coating	Surface	Animal Model Used	Mechanism of Action	Reference
Triclosan–Dispersin B (DspB)	Vascular catheter	Rabbit	Triclosan antibacterial activity, DspB biofilm matrix degradation, Biofilm dispersal, Anti-colonization, Sustained antimicrobial effect	[100]
MoS_2_–Ag_3_PO_4_	Titanium rib fixation plate	Rabbit	Silver ion antibacterial activity, MoS_2_ photothermal effect (NIR); ROS generation, Anti-biofilm, Anti-inflammatory, Osteogenesis promotion	[101]
SAAP peptides–PLEX (polymer–lipid matrix)	Subcutaneous implant	Mouse	Antimicrobial peptide membrane permeabilization, Anti-biofilm activity, Sustained controlled release, Anti-colonization, Activity against multidrug-resistant bacteria	[102]
Tannic acid–Ag nanoparticles–hydrophobic PFDT (TA–Ag–PFDT, LBL coating)	Silicone urinary catheter	Mouse, Rabbit	Silver ion antibacterial activity, Tannic acid antimicrobial effect, Hydrophobic anti-adhesion, Membrane disruption, Anti-biofilm; Reduced bacterial attachment	[103]
CuO–Ag nanoparticle–silk fibroin–polydopamine (pH-responsive composite coating)	Porous PEEK bone implant	Rabbit (tibia defect model)	pH-controlled Cu^2+^ and Ag^+^ release, High-dose antibacterial membrane disruption and anti-biofilm, Low-dose osteogenesis (ALP, collagen, mineralization), Angiogenesis (NO production), Osseointegration enhancement	[104]
PU–PDA–heparin–carboxymethyl chitosan (PU/PDA-Hep/CMCS)	Polyurethane implant	Rabbit	Heparin anticoagulant activity, Carboxymethyl chitosan antibacterial and anti-biofilm, Polydopamine adhesion layer, Hemocompatibility improvement, Anti-adhesion, Surface biofunctionalization	[105]
Vanillin–calcium phosphate	Calcium phosphate bone scaffold	Rabbit (New Zealand female)	Vanillin antimicrobial activity, Bactericidal and bacteriostatic effect against *Staphylococcus epidermidis*, Surface functionalization, Anti-colonization, Maintained osteointegration, Biocompatible coating	[106]
Ag nanoparticle–TiO_2_ nanotube–vancomycin	Titanium implant with TiO_2_ nanotubular orthopedic surface	Rabbit	Ag nanoparticle contact-killing, Vancomycin release killing; Anti-biofilm, MRSA membrane disruption; Dual antibacterial (contact release), Fibroblast-assisted antibacterial effect	[107]
MOX–SIM@ZIF-8–PDA coating	Polyetheretherketone (PEEK) orthopedic implant	Rat	Moxifloxacin antibacterial burst release, Simvastatin osteogenic activation, Zn2+ osteogenesis and antimicrobial support, Anti-biofilm activity; PDA-mediated adhesion and coating stability, Sustained dual drug release, Enhanced osseointegration	[108]
CarboCell hydrogel depot + levofloxacin/clindamycin ± cis-2-decenoic acid/cis-11-methyl-2-dodecenoic acid	Implant-associated osteomyelitis model	Rat, Pig	Sustained local antibiotic release, High-dose in situ drug delivery, Anti-biofilm fatty acid signaling disruption, Bacterial eradication, Infection clearance in bone and implant sites	[109]

## Data Availability

No new data were created or analyzed in this study. Data sharing is not applicable to this article.

## Abbreviations

NPs	Nanoparticles
AMR	Antimicrobial resistance
EPS	Exopolysaccharides
MRSA	Methicillin-resistant *Staphylococcus aureus*
HGT	Horizontal gene transfer
OMVs	Outer membrane vesicles
ROS	Reactive oxygen species
TPLO	Tibial plateau leveling osteotomy
